# Microsaccade Dynamics During Visual Fixation as Markers for Parkinson's Disease: A Machine Learning Approach

**DOI:** 10.1167/tvst.15.6.19

**Published:** 2026-06-16

**Authors:** Yiting Wang, Panagiota Tsitsi, Ioanna Markaki, Per Svenningsson, Gustaf Öqvist Seimyr, Tony Pansell, Mattias Nilsson

**Affiliations:** 1 Department of Clinical Neuroscience, Eye and Vision, Karolinska Institutet, Stockholm, Sweden; 2 Department of Clinical Neuroscience, Neuro, Karolinska Institutet, Stockholm, Sweden; 3 Center for Neurology, Academic Specialist Center, Stockholm, Sweden

**Keywords:** Parkinson's disease (PD), eye movements, microsaccades, visual fixation, machine learning

## Abstract

**Purpose:**

Microsaccades have long been of interest in vision and oculomotor research, but their potential as biomarkers in movement disorders such as Parkinson's disease (PD) remains underexplored. This study investigates the extent to which microsaccades carry information that can differentiate individuals with PD from a healthy population.

**Methods:**

We analyzed eye movements from 50 individuals with early-to-moderate stage PD and 43 healthy controls (HCs) recorded during a visual fixation task, without physically restricting head movements or interfering with the participants’ natural behavior. To assess the predictive value of microsaccades for distinguishing PD from HCs, we train and evaluate a diverse set of machine learning classifiers on microsaccade features.

**Results:**

Microsaccades identified under these recording conditions show characteristics consistent with those reported in more controlled experimental settings. Microsaccades in the PD group occur more frequently, have larger amplitudes, and exhibit stronger horizontal bias than those in the HC group. Most classification models perform significantly better than chance. A polynomial support vector machine (SVM) achieves a classification accuracy of 77.4% on held-out test subjects, with balanced sensitivity (76.1%) and specificity (78.9%). Moreover, by discarding predictions with low confidence at the microsaccade level, performance can be further improved at the subject level.

**Conclusions:**

Microsaccades carry discriminative information capable of distinguishing individuals with PD from HCs, underscoring their potential value as early markers of motor dysfunction in PD.

**Translational Relevance:**

This study establishes a promising baseline against which future learning algorithms can be evaluated, with the long-term goal of developing clinically useful prediction models based on oculomotor behavior.

## Introduction

The progression of Parkinson's disease (PD) is associated with neurological changes that adversely affect eye movements and oculomotor control.^1^^–^^5^ Research in this area has often focused on how PD affects larger, voluntary, and cognitively guided eye movements, making it easy to overlook the fact that our eyes are constantly moving, regardless of intention or cognitive engagement. Even when we simply try to keep our eyes steady on a stationary target, our gaze is continuously adjusted by small, involuntary eye movements of which we are generally unaware. Such fixational eye movements are part of the normal functioning of the oculomotor system and include ocular tremor, drift, and microsaccades.^6^

Microsaccades, the largest type of fixational eye movements, occur approximately once or twice per second with amplitudes typically less than half a degree of visual angle. These small eye movements have been of interest to vision scientists and oculomotor researchers since the 1950s, partly because their occurrence does not rely on conscious intent and partly because their functional significance has proven difficult to explain.^7^^–^^15^ Over the years, accumulating evidence has indicated that microsaccades play an important role in visual perception, helping to counteract neural adaptation and prevent perceptual fading of unchanging visual stimuli.^6^^,^^16^^,^^17^ As such, microsaccades provide a window into visuomotor control at a microscopic scale, enabling investigation of the dynamics of the smallest saccadic eye movements and their effects on vision in both healthy and clinical populations.^18^^,^^19^

To date, little is known about the extent to which microsaccades carry information that differentiates individuals with PD from a healthy population. One reason for this is the absence of systematic investigations using predictive modeling techniques grounded in machine learning methodology, with well-defined training and testing procedures for model validation. Whereas conventional statistical methods focus on group-level inference based on parameter estimation using all available data, machine learning methods aim to identify patterns that generalize to new individuals by using separate training and evaluation datasets to assess predictive performance. This makes machine learning a powerful approach for investigating whether microsaccade dynamics can serve as sensitive markers of PD at the individual level.

In the present study, we analyze eye movement data from a simple visual fixation task in which 50 individuals with early-to-moderate stage PD (Hoehn and Yahr^20^ stages 1–3) and 43 healthy participants were instructed to fixate on a small central fixation target. To avoid interfering with the natural behavior of the participants, no head-stabilization equipment (such as a bite-bar or chin-and-forehead rest) was used in these experiments. This was in keeping with the objectives of a larger research project investigating eye tracking in neurodegenerative diseases under minimally constrained and naturalistic recording conditions.

To assess the overall validity of the data for microsaccade analysis, we first evaluate the quality of the eye-tracking signals, examine the presence of head movements, and determine the extent to which detected saccades exhibit properties that are characteristic of microsaccades, including their frequency, amplitude, and direction. Next, we assess whether participants with PD differ from healthy controls (HCs) in microsaccade characteristics using robust linear mixed-effects models, including participants as random effects with random intercepts to account for individual variability. Finally, to assess the extent to which microsaccade dynamics can predict PD at the individual level, we train and evaluate a set of well-established classification algorithms on the microsaccade data. By applying a diverse set of models with distinct underlying principles, our goal is to establish a performance baseline against which more complex methods and learning algorithms can be systematically evaluated in future work.

## Materials and Methods

### Participants and Clinical Assessment

A total of 93 individuals participated in the study, including 50 participants diagnosed with PD (66% male participants) and 43 healthy age-matched controls (37% male participants), with an overall mean age of 63.1 years (SD = 9.3). The diagnosis was based on the United Kingdom Parkinson's Disease Society (UKPDS) Brain Bank Criteria.^21^ PD participants at Hoehn and Yahr stages 1 to 3 were enrolled through the Center of Neurology at the Academic Specialist Center in Stockholm, Sweden. Clinical severity of PD was assessed with the Unified Parkinson's Disease Rating Scale (UPDRS).^22^ In addition, both PD and HC participants completed three cognitive screening assessments: the Montreal Cognitive Assessment (MoCA),^23^ the Mini-Mental State Examination (MMSE),^24^ and the Frontal Assessment Battery (FAB).^25^ Clinical assessments were conducted while PD participants were on their regular medication regimen.

The median time since diagnosis among PD participants was 2 years, with a median Levodopa Equivalent Daily Dose (LEDD)^26^ of 545 mg/day, and a median score of 21 on the motor subsection of the UPDRS-III (max = 108). On the Schwab and England Activities of Daily Living (ADL)^27^ scale, the median score was 90%, indicating a high level of functional independence. Overall, these clinical characteristics are consistent with early-stage PD, although the degree of impairment varied among individuals.

Cognitive assessments revealed no statistically significant differences between the PD and HC groups in total scores on the MoCA, MMSE, or FAB. Relevant to the sustained fixation task, there was also no significant group difference in the MoCA attention subdomain, which includes an auditory vigilance task to assess sustained attention.

The PD group, however, performed significantly worse in the visuospatial/executive subdomain of the MoCA. This domain includes tasks such as drawing a clock and copying a three-dimensional figure, which assess visuospatial reasoning, executive planning, and visuomotor (eye-hand) coordination.

To ensure adequate visual function, individuals with macular degeneration or untreated cataracts were not included in the study. One HC participant and three participants with PD had glaucoma; in all cases, the condition was treated, well controlled, and did not preclude participation in the study. All participants had normal or corrected-to-normal vision.

A more detailed description of study participants and their clinical characteristics can be found in a previous publication*.*^28^

The study received ethical approval from the regional ethical review board in Stockholm (DNR: 2018/437-31) and adhered to the principles of the Declaration of Helsinki. Written and verbal informed consent was obtained from all participants.

### Experimental Setup

Eye movements were recorded with a Tobii Pro Spectrum, a screen-based eye tracker designed for detailed studies of natural human behavior and the mechanics of the fastest eye movements. According to the manufacturer, it tolerates more head movements than any other high-frequency screen-based eye tracker on the market.^29^ Data were recorded binocularly, capturing gaze positions for both left and right eyes at the maximum sampling rate of 1200 hertz (Hz). Stimuli presentations were created using the software platform Tobii Pro Lab and displayed on the integrated Tobii Pro Spectrum monitor (23.8” EIZO FlexScan EV2451) with a resolution of 1920 × 1080 pixels (52.8 × 29.7 cm).

Participants were seated on a stable chair in a dimly lit room, approximately 60 cm from the screen, positioning themselves such that the average position of their eyes aligned with the center of the eye tracker's headbox. To minimize potential discomfort associated with obtrusive or movement-limiting equipment in the clinical group, recordings were performed without head-stabilization, in keeping with the main project's emphasis on ecological validity. However, although the recording setup allowed head movement in principle, the fixation task constrained head movement in practice. This is particularly apparent when contrasted with head-free viewing paradigms involving free exploration of dynamic scenes, rather than prolonged fixation of a small, stationary central target.

Studies of microsaccades that do not involve head-stabilization are rare but do exist, particularly in research emphasizing ecologically valid settings and real-world scenarios.^30^ For example, a number of studies on eye movements during driving have explored the relationship between microsaccades and factors such as driver fatigue and road distractions.^31^^–^^34^

A five-point calibration was performed, with re-calibration initiated if visual inspection revealed significant deviation in one or more calibration points. Prior to the fixation task, a brief assessment of visual acuity was conducted using a digitized Landolt “C” eye chart to ensure participants could reliably resolve the fixation stimulus. Participants were then instructed to fixate on a small black dot, measuring 6 mm in diameter and subtending approximately 0.5 degrees of visual angle. The fixation target was presented centrally against a white background. Participants were not instructed to refrain from blinking, to avoid the fixation task inadvertently becoming an exercise in blink suppression. All participants completed 8 fixation trials, each lasting 15 seconds, with a 5-second intertrial interval. This resulted in 120 seconds of attempted visual fixation per participant, corresponding to approximately 3 hours of fixation data across all participants.

### Preprocessing and Microsaccade Detection

Eye movement recordings were exported from Tobii Pro Lab as raw data and processed as follows. First, blinks and longer track loss segments were identified and excluded. These segments were detected based on periods of missing samples in the pupil size signal, where any “gap” exceeding 10 ms was classified as a potential blink segment. If 2 or more such segments occurred within 25 ms of each other, they were merged along with any intervening samples into a single blink period. This process was repeated until no further segments could be merged. Each such period was then extended by 30 ms at both ends to capture any residual tracking instability. Any remaining brief episodes of track loss that were not recorded as part of a blink were replaced using linear interpolation.

Gaze positions in pixel coordinates were converted to degrees of visual angle and low-pass filtered using a 20 ms Bartlett window. A previous study using the same eye tracker demonstrated that this filter and window size produced stable and plausible microsaccade rates across four participants during a sustained fixation task.^35^ A commonly used algorithm for microsaccade detection^36^ was then applied to the filtered data. This algorithm identifies microsaccades as outliers in two-dimensional velocity space based on a velocity threshold that adapts to the level of noise in the data. Detection was performed using the default threshold, defined as six times the standard deviation (SD) of the median velocity (λ = 6). To further reduce noise and minimize detection errors, three additional criteria were applied. First, to prevent dynamic overshoots from being classified as separate saccades, any saccade detected within 10 ms of a preceding saccade was treated as part of the initial saccade and merged into a single saccade event. Second, to avoid mistaking brief transient noise for microsaccades, a minimum duration threshold of 6 ms was applied. Finally, in line with the general view that microsaccades are binocular phenomena,^17^^,^^37^^,^^38^ only saccades with temporal overlap in both eyes (i.e. binocular saccades) were counted as valid microsaccades.

It is a common approach to apply cutoff thresholds to limit the possible physical extent of microsaccadic parameters, for example, excluding saccades with amplitudes greater than 1 degree or peak velocities exceeding 100 degrees/s. However, in a clinical context, such fixed thresholds may obscure meaningful differences in microsaccade characteristics between patient and control groups. To avoid imposing strict, predefined limits, we applied a Hampel filter to the amplitude and velocity distributions within each group, identifying outliers as values exceeding three median absolute deviations from the group median. This approach accommodates potential inter-group variability by avoiding hard cutoffs, while still excluding extreme data points that deviate markedly within each group. After filtering, 88.4% of saccade detections remained in the PD group, corresponding to 4714 microsaccades from 46 participants. In the HC group, 86.2% of saccade detections were retained, totaling 3924 microsaccades from 38 participants.

For simplicity, we refer to all saccadic eye movements in our analysis as microsaccades, or simply saccades, regardless of their size or other physical characteristics, unless otherwise specified. This usage is consistent with an operational definition of microsaccades as involuntary saccades occurring during attempted fixation.^18^ We further note that no attempt was made to separately identify or classify saccadic intrusions, such as square-wave jerks, from other saccades.

### Statistical Analysis

When summarizing data at the group level, we primarily report statistics that do not assume normality, presenting medians and interquartile ranges (IQRs) in most cases. Based on visual inspection of the data, nonparametric measures better capture central tendency and reduce the influence of extreme values, which can skew mean estimates and potentially drive significant effects. Analyses with robust linear mixed effects models were conducted in R software (version 4.4.2) using the packages *robustlmm* (version 3.3–1)^39^ and *boot* (version 1.3–31).^40^ *P* values and 95% confidence intervals (CIs) for fixed effects (group differences) were estimated via nonparametric bootstrapping with 1000 resamples. CIs were derived using the percentile method, and *P* values were calculated as the proportion of bootstrap samples in which the group effect exceeded or fell below zero, corresponding to a two-tailed test.

### Machine Learning

Ten basic machine learning algorithms were evaluated to establish baseline performance. The selected algorithms span several classes of machine learning models, including linear classifiers, tree-based methods, ensemble approaches, instance-based methods, probabilistic models, support vector machines, and neural networks, enabling comparisons across different modeling approaches. The specific models included in this work are Adaptive Boosting (AdaBoost), Bootstrap aggregating (Bagging), Random Forest (RF), Decision Tree (DT), Logistic Regression (LR), K-nearest neighbor (kNN), Naïve Bayes (NB), Multilayer perceptron (MLP), Support Vector Machine with a polynomial kernel (SVM-Poly), and Support Vector Machine with a linear kernel (SVM-Linear) (see Supplementary Material for a brief description of each model).

All models were trained using the caret package in R software. All experiments were evaluated using leave-one-subject-out (LOSO) cross-validation. This resampling strategy maximizes the amount of data used for training while ensuring that the test data are entirely independent from the training data, such that model performance reflects generalization to unseen individuals. The model was iteratively trained on the microsaccades of all subjects except one, whose data were excluded from the training set. In each iteration, the model was tested on the microsaccades of the currently held-out subject, yielding predictions for all individual microsaccades of that subject. In our experimental setup, the total number of subjects was 84 (i.e. 83 were used for training and 1 for testing in each fold). The number of microsaccades naturally varied between subjects, but the average number of microsaccades per fold was approximately 8500 in the training set and 100 in the test set.

Furthermore, we hypothesized that discarding predictions with low confidence and high uncertainty at the microsaccade level may improve overall classification performance at the subject level. Therefore, we conducted a set of experiments systematically removing predictions with low confidence. Low-confidence predictions were defined as those for which the predicted probability of the positive class (PD) fell within the interval 0.5 ± α. A threshold of α = 0.05, for example, excluded predictions with probabilities in the range 0.45 to 0.55. We evaluated 5 thresholds (α = 0.05–0.25, step size = 0.05), corresponding to progressively wider symmetric exclusion intervals centered at 0.5.

Performance was assessed using standard classification metrics including accuracy, along with sensitivity and specificity, as well as the area under the receiver operating characteristic curve (ROC-AUC). Sensitivity refers to the model’s ability to correctly identify individuals with PD (true positive rate), whereas specificity measures its ability to correctly rule out PD, that is, to correctly identify HC (true negative rate).

Although all models operate at the microsaccade level, predictions were aggregated at the subject level using soft voting, reflecting the greater clinical relevance of distinguishing between healthy individuals and individuals with PD rather than classifying individual microsaccades.

### Code Accessibility

Code for the machine learning experiments may be obtained from the corresponding author upon reasonable request.

## Results

### Data Loss and Exclusion

Data from six participants (3 with PD and 3 HCs) were entirely excluded from analysis, either due to calibration failures, excessive interruptions in the tracking signal, or other technical issues. In addition, partial data from recordings of nine PD participants and six HC participants were discarded. Specifically, trials containing no valid gaze samples after filtering out blinks and longer signal disruptions were excluded (29 PD trials and 19 HC trials). Consequently, data from 87 participants (47 with PD and 40 HCs) were included in the analysis, encompassing a total of 648 trials, with 347 PD trials (86.8% of all original PD trials) and 301 HC trials (87.5% of all original HC trials). The sex ratio remained essentially unchanged in both groups after filtering, with 65% male patients with PD (originally 66%) and 39% male HCs (originally 37%). Median age did not differ significantly between groups in the filtered dataset (PD = 64 years and HC = 63 years; Wilcoxon rank-sum test: *W* = 844, *P* = 0.79), consistent with the original data.

**Figure 1.**
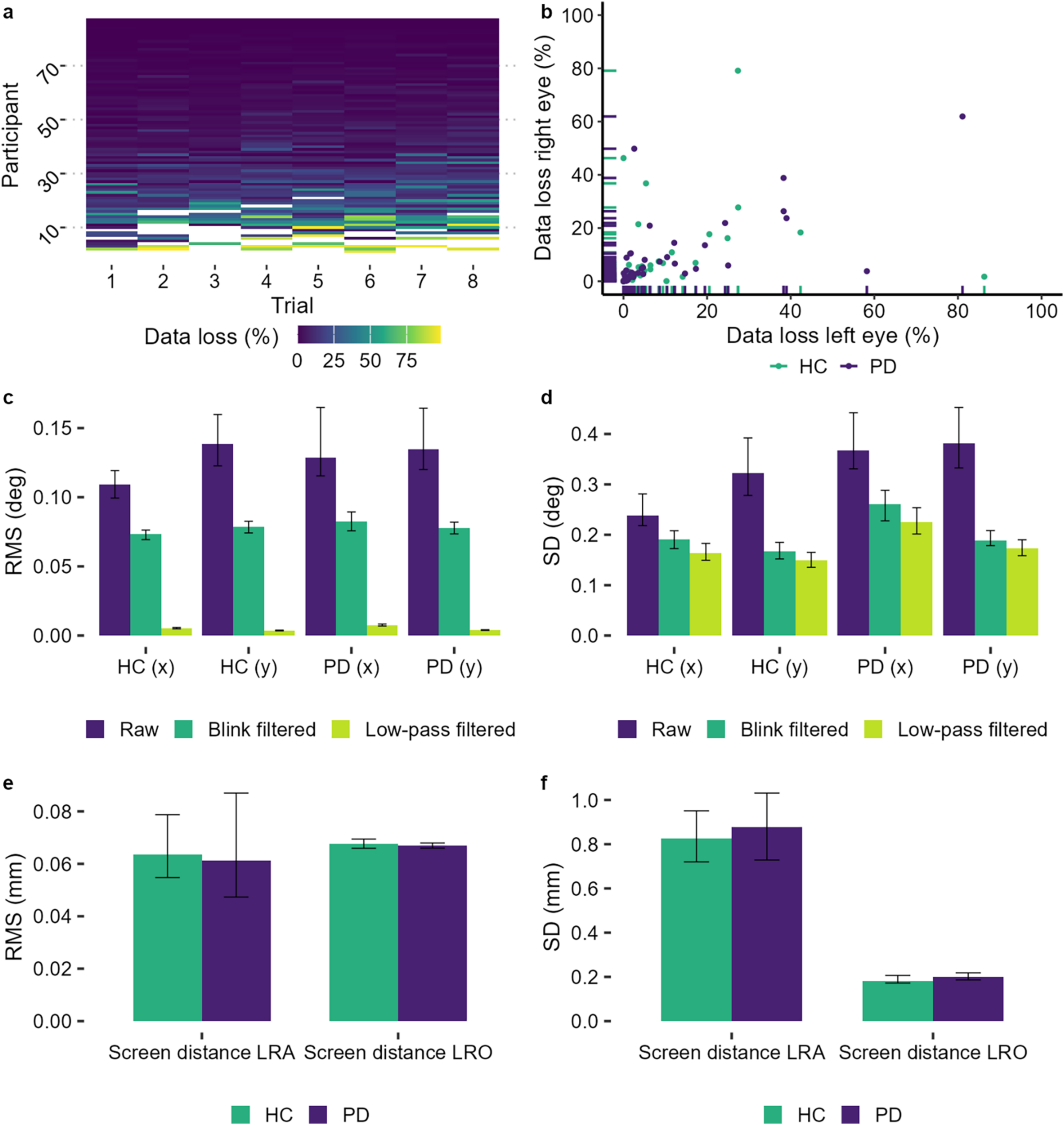
**Data quality: sample loss, gaze precision, and head movements.** (**a**) Heatmap of sample loss per trial for each participant, sorted by mean loss (participants with the highest loss appear at the *bottom*). Each *row* corresponds to a participant, and each *column* to a trial. Color indicates the percentage of data loss, calculated as the proportion of gaze samples missing data from one or both eyes. *White cells* represent excluded trials (100% data loss). (**b**) Scatter plot of left- and right-eye sample loss for each participant by group (HC and PD). Each point corresponds to the average percentage of sample loss across available trials. (**c,** **d**) Root-mean-square (RMS) of inter-sample distances **c** and standard deviation (SD) **d** of horizontal (x) and vertical (y) gaze signals for raw, blink filtered, and low-pass filtered gaze data in each group. (**e,** **f**) Root-mean-square (RMS) of inter-sample distances **e** and standard deviation (SD) **f** of eye-to-screen distance measures, used as proxies for head movements: left-right average (LRA) and left-right offset (LRO). Bars in **c** to **f** represent the median across trials; error bars show 95% confidence intervals of the median estimated via bootstrapping (1000 resamples).

A trial-by-trial heatmap profile of data loss across the participants shows that most missing gaze data originated from a minority of participants, with 10 individuals accounting for 50% of the total loss (Fig. 1a). Most participants exhibited low and symmetrical data loss between the left and right eyes, consistent with natural blink behavior, although clear exceptions were also observed (Fig. 1b). Across trials, data loss in the left and right eyes was strongly correlated (Spearman's rank correlation: *r* = 0.74, *P* < 0.05), and neither eye was significantly more prone to data loss than the other (Wilcoxon signed-rank test: *V* = 92,952, *P* = 0.51). The median data loss per trial was 3.1% in the HC group (IQR = 0.9%–11.3%) and 3.7% in the PD group (IQR = 0.6%–13.6%), calculated as the proportion of gaze samples missing data from one or both eyes. The observed difference between the groups was not statistically significant (Wilcoxon rank-sum test: *W* = 51,443, *P* = 0.74).

### Precision

Because microsaccades occur over only a few data samples even at high sampling rates (e.g., at 1200 Hz, a microsaccade with a duration of 20 ms spans only 24 data points), reliably detecting and distinguishing them from background noise or recording artifacts requires gaze data with high precision, characterized by low variable error. To quantify the magnitude of variability in the gaze signals, we computed two complementary metrics that are standard in the field: the root mean square (RMS) of inter-sample distances between consecutively valid gaze samples, which captures the extent of rapid, moment-to-moment fluctuations in the signal; and the SD of gaze position, which reflects the overall dispersion of gaze samples around the center of fixation. The results for raw, blink-filtered, and low-pass filtered data are shown in Figures 1c and 1d. It is important to note that whereas RMS and SD are commonly used to quantify noise or signal precision in eye-tracking systems under ideal conditions (e.g., using artificial eyes), variability in the gaze signal during a human fixation task reflects not only measurement noise, but also real oculomotor behavior. In this context, elevated RMS or SD values may reflect inherent fixation instability in participants, potentially due to more frequent microsaccades, occasional saccadic intrusions, or generally more dispersed and unstable fixation.

In the raw gaze data, the median RMS across horizontal and vertical gaze signals was 0.12 degrees (IQR = 0.08 degrees to 0.26 degrees) in the HC group and 0.13 degrees (IQR = 0.08 degrees to 0.33 degrees) in the PD group. The median SD of gaze position was 0.28 degrees (IQR = 0.16 degrees to 0.56 degrees) in the HC group and 0.38 degrees (IQR = 0.20 degrees to 0.71 degrees) in the PD group. The difference in inter-sample RMS between groups approached significance (*W* = 196,020, *P* = 0.055), and the SD of gaze position was significantly higher in the PD group (*W* = 179,817, *P* < 0.001), as assessed by the Wilcoxon rank-sum test.

We observed that RMS values in the raw data were strongly correlated with the proportion of missing gaze samples per trial (Spearman's rank correlation: *r* = 0.74, *P* < 0.05) suggesting that the sample-to-sample variability was closely associated with the amount of data loss. Because RMS is computed only between consecutively valid gaze samples – explicitly excluding gaps due to blinks – this finding indicates that blink-related artifacts contributed substantially to the variability in the raw signal. This is not unexpected, as blinks typically introduce signal instability when the eyelids close and reopen and the pupil is partially occluded, making tracking less reliable. Such issues are also likely to increase with the age of participants, due to age-related changes in eyelid physiology or tear film stability that can affect blinking behavior. As anticipated, removing blinks and surrounding artifacts substantially improved precision and reduced the differences between the groups.

Low-pass filtering the signals further reduced gaze variability to levels comparable to those typically expected for microsaccade analysis, with a mean RMS of 0.01 degrees and a mean SD of 0.23 degrees after excluding extreme outliers (defined as values beyond Q1 − 3 × IQR or Q3 + 3 × IQR). As expected, low-pass filtering had comparatively little effect on SD, consistent with the fact that SD reflects the overall spread of gaze samples in the signal, rather than the extent of high-frequency fluctuations.

### Head Movements

Although the eye-tracking system maintains accurate gaze estimates despite natural changes in head position, excessive head movements may still be detrimental to signal quality and microsaccade detection. To address this concern, we assessed the variability in head position during the fixation task. Changes in eye-to-screen distance were analyzed as a proxy for head motion along the depth axis, reflecting a combination of forward-backward translation and vertical head rotation (pitch). In addition, the difference between the left and right eye-to-screen distances served as a proxy for horizontal head rotation (yaw). A negative offset indicates that the left eye was closer to the screen than the right eye, corresponding to a rightward head rotation, whereas a positive offset indicates that the right eye was closer than the left, corresponding to a leftward rotation. For both proxy signals, we computed the inter-sample RMS to quantify moment-to-moment fluctuations, and the SD to capture overall variability in head position within each trial (Figs. 1e, 1f).

Overall, the extent of head movements was comparable between the two groups. The median inter-sample RMS of eye-to-screen distance across trials was 0.06 mm in both groups (IQR = 0.04–0.16 mm in HCs and 0.04–0.20 mm in patients with PD). At the eye tracker sampling rate (1200 Hz), this corresponds to an angular velocity of approximately 0.20 degrees/s, assuming uncorrelated sample-to-sample changes at a 60 cm viewing distance. The median SD of eye-to-screen distance across trials was 0.83 mm in the HC group (IQR = 0.43–1.76 mm) and 0.88 mm in the PD group (IQR = 0.44–1.81 mm), indicating that participants maintained a stable overall head position, with only small deviations from their average distance to the screen within each trial. No significant group differences were observed for either the RMS (*W* = 51,613, *P* = 0.80) or the SD (*W* = 51,706, *P* = 0.83) of eye-to-screen distance, as assessed by the Wilcoxon rank-sum test. The PD group appeared to show greater variability in eye-to-screen distance, as reflected by wider CIs for both RMS and SD. However, on closer inspection, this difference disappeared when extreme outliers were excluded.

The median inter-sample RMS of left–right eye-to-screen offset across trials was 0.07 mm in both groups (IQR = 0.06–0.09 mm in HCs and 0.06–0.08 mm in patients with PD). At the eye tracker sampling rate, this corresponds to an angular velocity of approximately 0.22 degrees/s, assuming uncorrelated sample-to-sample changes. The median SD of left–right offset was 0.18 mm in the HC group (IQR = 0.14–0.35 mm) and 0.20 mm in the PD group (IQR = 0.15–0.37 mm), reflecting minimal overall variability in horizontal head rotation. Notably, the SD of horizontal head rotation was markedly lower, in relative terms, than that of movements along the depth axis. A plausible explanation for this difference is that participants maintained a consistently centered head orientation over time, resulting in minimal left–right deviation. In contrast, subtle postural adjustments associated with breathing likely caused slow forward–backward head movements along the depth axis, contributing to a comparatively larger SD. No significant group differences were observed for either the RMS (*W* = 53,817, *P* = 0.50) or the SD (*W* = 48,250, *P* = 0.10) of eye-to-screen left–right offset, as assessed by the Wilcoxon rank-sum test.

Based on the inter-sample RMS with the largest interquartile spread observed in our data (1.39–6.93 mm/s), corresponding to approximately 0.13 to 0.66 degrees/s at a 60 cm viewing distance, head movements appear too slow to produce gaze shifts that could interfere with the detection of microsaccades. Although the angular displacement is similar to that of microsaccades, the time course is very different. Microsaccades typically occur over 10 to 30 ms, reaching velocities of 15 to 50 degrees/s, whereas motion of the head in our data occurs at a far slower pace, with estimated angular fluctuations typically below 1 degree/s.

### Microsaccade Characteristics

**Figure 2.**
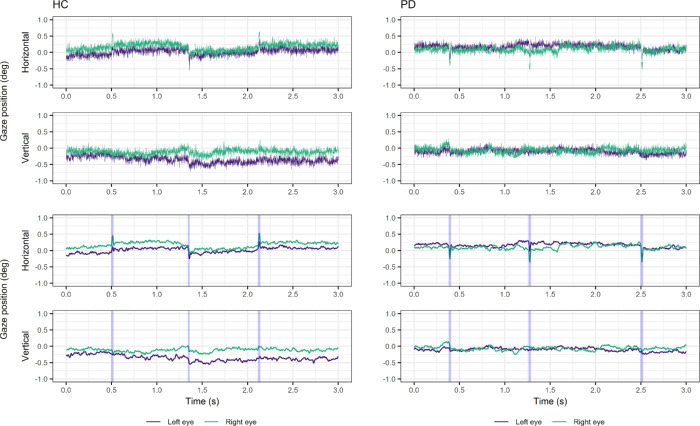
**Gaze position over time with detected microsaccades.** Horizontal and vertical gaze signals from the left and right eyes over a 3-second period, shown for raw (*top*) and filtered (*bottom*) data for 2 study participants. *Lightly shaded vertical bars* mark events identified as binocular microsaccades using a commonly applied velocity-based algorithm.^36^ *Left*: Healthy control. *Right*: Participant with Parkinson's disease.

Figure 2 shows horizontal and vertical gaze signals recorded from the left and right eyes over a 3-second period for one HC and one PD participant. The sudden changes that are only weakly visible in the horizontal components of the raw signals are identified as binocular microsaccades by the detection algorithm when applied to the filtered data and are highlighted by shaded vertical bars in the graph. A number of characteristics commonly attributed to microsaccades can be observed in these examples selected here for illustrative purposes. Microsaccades are typically reported to occur at a rate of 1 to 2 Hz. Here, 3 microsaccades were detected for each of the participants, with a mean inter-saccadic interval of approximately 850 ms, corresponding to a frequency of approximately 1.2 Hz. In line with the common observation that microsaccades are more prevalent in the horizontal direction, it can also be observed that the microsaccades are more pronounced in the horizontal component of the gaze signal than in the vertical component. This is true for both participants, although the tendency is more noticeable in the PD participant, where vertical displacement is minimal. With respect to the size of microsaccades, we observe that they are indeed small, with amplitudes not exceeding half a degree of visual angle in these examples. Furthermore, we also observe a slight difference in microsaccade amplitude between the two participants, with those of the PD participant being slightly larger than those of the HC participant.

**Figure 3.**
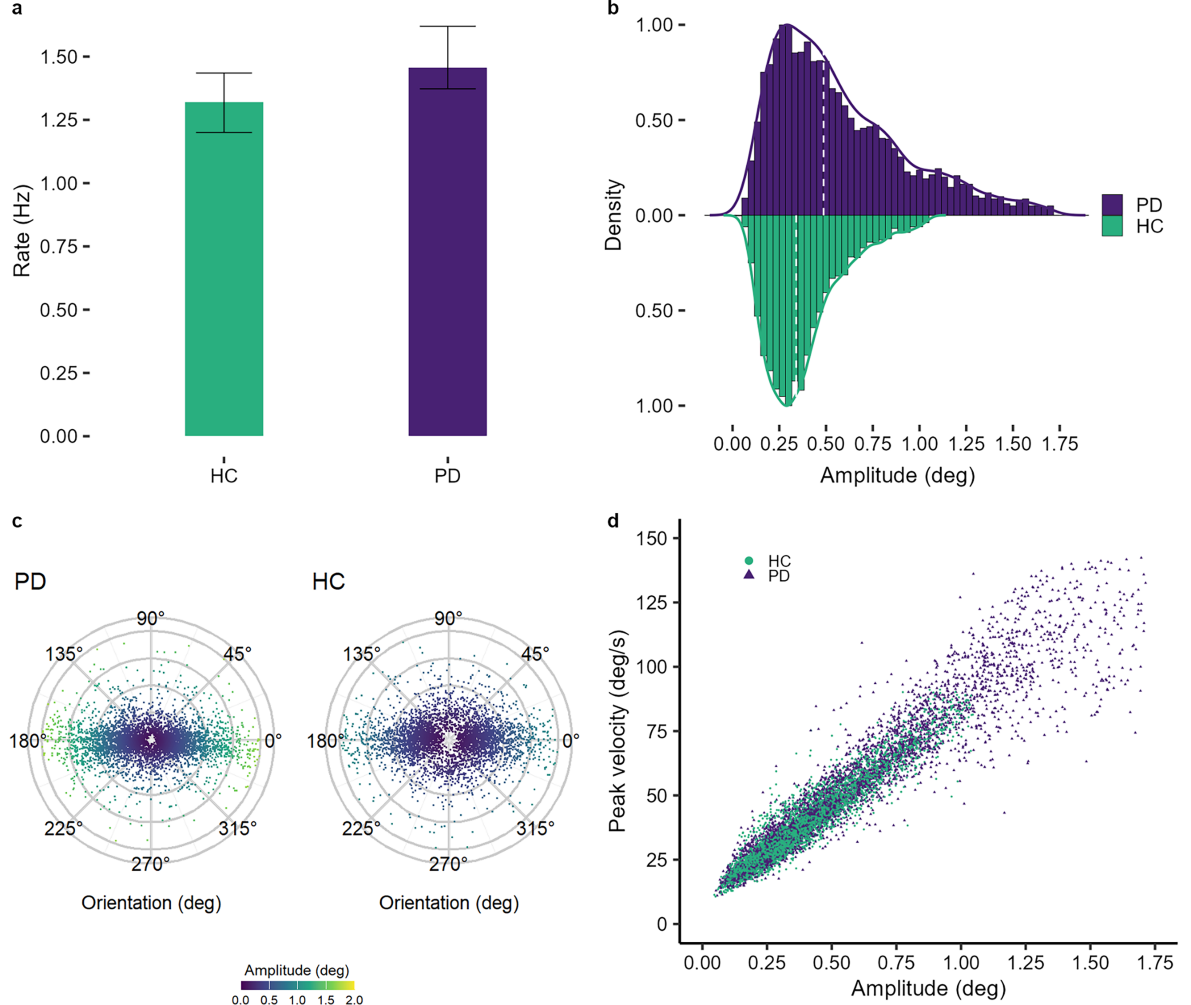
**Microsaccade characteristics by participant group (HC** **and** **PD).** (**a**) Microsaccade rate. Rate was calculated by dividing the number of microsaccades in a trial by the total duration of valid samples in the trial. *Bars* represent the median across trials, with error bars showing 95% confidence intervals of the median, estimated via bootstrapping (1000 resamples). (**b**) Microsaccade amplitude. Mirrored histograms showing the distribution of microsaccade amplitudes in each group. Amplitude was calculated as the maximum distance between any two samples within each detected microsaccade. The x-axis represents amplitude, and the y-axis shows relative density, normalized such that the tallest bin in each group is scaled to 1. *White dashed vertical lines* indicate the median amplitude for each group. **(c**) Microsaccade orientation. Polar plots showing microsaccade orientation (angle) and amplitude (radial distance). Each *dot* represents one microsaccade. The outermost circle corresponds to the maximum amplitude within each group, and the spacing is scaled proportionally to each group's maximum amplitude. Because amplitude scaling is group-specific, cross-group comparisons should rely on color rather than radial distance, with lighter colors indicating larger amplitudes. Microsaccades to the right are at 0 degrees, and upward movements are at 90 degrees. (**d**) Microsaccade main sequence. Scatter plot of peak velocity as a function of amplitude. Each *dot* represents a single microsaccade.

Figure 3 summarizes group-level microsaccade characteristics based on a total of 8638 detected microsaccades, focusing on the quantitative properties most commonly reported in the literature. Overall, the detected microsaccades exhibited properties consistent with previous findings in terms of their frequency of occurrence, amplitude distribution, orientation bias, and amplitude-velocity relationship. The median microsaccade rate was slightly higher in the PD group (1.5 Hz, IQR = 0.9–2.3 Hz) compared to the HC group (1.3 Hz, IQR = 0.9–1.8 Hz; see Fig. 3a). The median amplitude was also larger in the PD group (0.49 degrees, IQR = 0.30–0.77 degrees) than in the HC group (0.34 degrees, IQR = 0.24–0.48 degrees), reflecting a broader amplitude distribution and a greater proportion of larger microsaccades in PD (see Fig. 3b). More specifically, whereas 99.4% of microsaccades in the HC group were smaller than 1 degree, a commonly used upper cutoff for microsaccades, the corresponding proportion in the PD group was 86.4%. Microsaccades exhibited a strong horizontal bias in both groups, consistent with previous findings (see Fig. 3c). This horizontal predominance appeared slightly more pronounced in the PD group. A two-sample *z*-test for equality of proportions confirmed a significantly higher proportion of horizontal microsaccades (defined as those with an orientation within ±22.5 degrees of 0 degrees or 180 degrees) in the PD group (62.9%) compared to HC group (59.2%), a difference of 3.7 percentage points (95% CI = 1.58 to 5.76, *P* < 0.001). Figure 3d shows the microsaccade main sequence, a well-known saccadic relationship in which peak velocity increases with saccade amplitude.^14^^,^^31^ To characterize the relationship, we fitted separate linear regression models for the HC and PD groups, predicting peak velocity from microsaccade amplitude. Both groups showed a strong linear relationship, as reflected in high *R²* values (HC = 0.897 and PD = 0.899). However, the residual standard error was notably larger in the PD group (8.6 degrees/s) compared to the HC group (5.0 degrees/s), indicating greater variability around the main sequence in PD. An F-test for equality of residual variances confirmed this difference (*F* = 0.34, *P* < 0.001), a result further corroborated by a Fligner–Killeen test (χ² = 340.85, *P* < 0.001).

Next, we fitted robust linear mixed-effects models comparing HC and PD participants across the parameter’s microsaccade rate, amplitude, and orientation bias. In these analyses, participants were included as random effects (with random intercepts) to account for individual variability and to ensure that group-level effects were not confounded by between-subject differences. The results (see Table 1) show that microsaccade frequency was significantly higher in the PD group compared to HC, with a difference of 0.219 Hz (*P* < 0.001), corresponding to an estimated mean rate of 1.50 Hz (95% CI = 1.40–1.64 Hz). Amplitude was also significantly larger in the PD group, with a difference of 0.123 degrees (*P* < 0.001) and an estimated mean amplitude of 0.47 degrees (95% CI = 0.45–0.48 degrees). Further, orientation bias differed significantly between groups: the PD group exhibited a stronger horizontal component (β = 0.016, *P* < 0.001) and a weaker vertical component (β = −0.042, *P* < 0.001) relative to HCs, suggesting reduced vertical movement in PD. CIs for all estimates from the robust linear mixed-effects models excluded zero.

**Table 1.** Linear Mixed-Effects Model Results for Group Differences (HC versus PD) in Microsaccade Rate, Amplitude, and Orientation Bias

	Microsaccades			
			Orientation Bias	
	Rate, Hz	Amplitude, Degrees	Horizontal	Vertical
Intercept, β₀	1.280	0.344	0.867	0.348
SE, β₀	0.115	0.020	0.005	0.006
Fixed effect, β_PD	0.219	0.123	0.016	−0.042
SE, β_PD	0.156	0.026	0.007	0.008
Boot. *P* value	<0.001	<0.001	<0.001	<0.001
Boot. 95% CI lower	0.118	0.107	0.007	−0.057
Boot. 95% CI upper	0.356	0.140	0.026	−0.028
Observations, *N*	582	8638	17276	
Subjects, *n*	84	84	84	

Microsaccade rate and amplitude were analyzed on log-transformed data, with estimates back-transformed to the original units (shown in the table) for ease of interpretation. Orientation bias (horizontal and vertical) is a unitless index ranging from 0 to 1 computed as the absolute value of the cosine and sine of microsaccade orientation (0–360 degrees), respectively. The intercept (β₀) corresponds to the estimated mean for the HC group, and β PD represents the estimated difference between PD and HC. Standard errors (SEs) are reported for both estimates. *P* values and 95% confidence intervals for estimated group differences were obtained via bootstrapping (1000 resamples).

**Figure 4.**
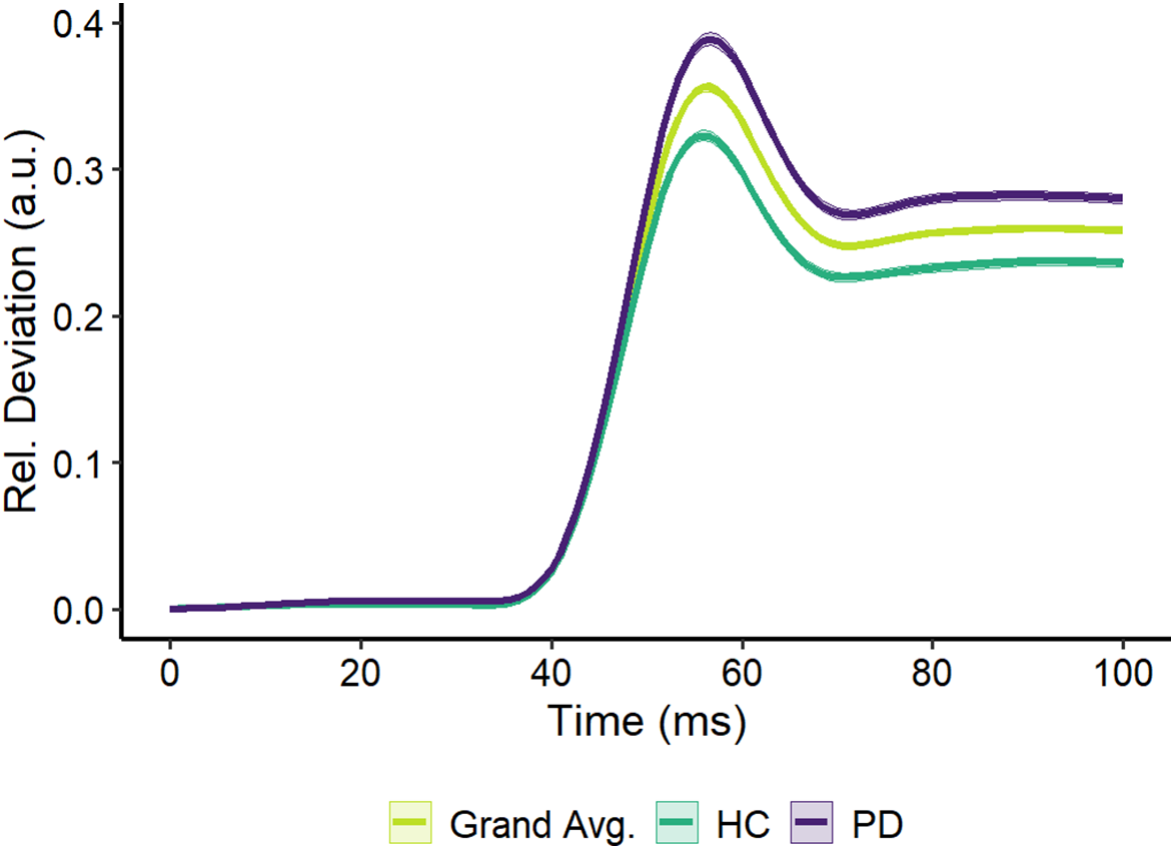
**Average microsaccade waveforms**. Average microsaccade waveforms for each participant group and the grand average across all participants. Each waveform is based on the horizontal gaze signals from all microsaccades with amplitudes up to 1 degree. The x-axis denotes time in milliseconds (ms) whereas the y-axis quantifies the relative deviation of the horizontal gaze signal in arbitrary units (a.u.) from the fixation point. Number of microsaccades per waveform: HC = 3971 and PD = 4039; Grand average = 8010.

Finally, to characterize the temporal profile of microsaccades, we computed an average waveform by extracting all microsaccades with amplitudes up to 1 degree, aligning them to saccade onset, and normalizing each waveform by its maximum absolute deviation from baseline. The resulting waveforms reflect the relative shape of the movement trajectory over time, independent of differences in amplitude across individual microsaccades. Figure 4 shows the average waveforms for HC, individuals with PD, and the grand average. The waveforms follow a well-defined, stereotyped time course consistent with previous descriptions of how microsaccades unfold over time. Whereas the temporal profiles are highly similar across the two groups, the PD waveform shows a larger relative deviation compared to HCs, likely reflecting a larger microsaccade amplitude on average.

### PD Classification

Classification models were trained at the microsaccade level to predict whether a given microsaccade was more likely to originate from a PD participant or an HC participant. To determine an overall prediction for each held-out test participant, microsaccade-level predictions were aggregated by calculating the mean probability for each class (HC and PD). The class with the highest mean probability was then assigned as the subject-level prediction. In this way, the predicted probabilities from each microsaccade contributed to the final classification of the participant.

**Figure 5.**
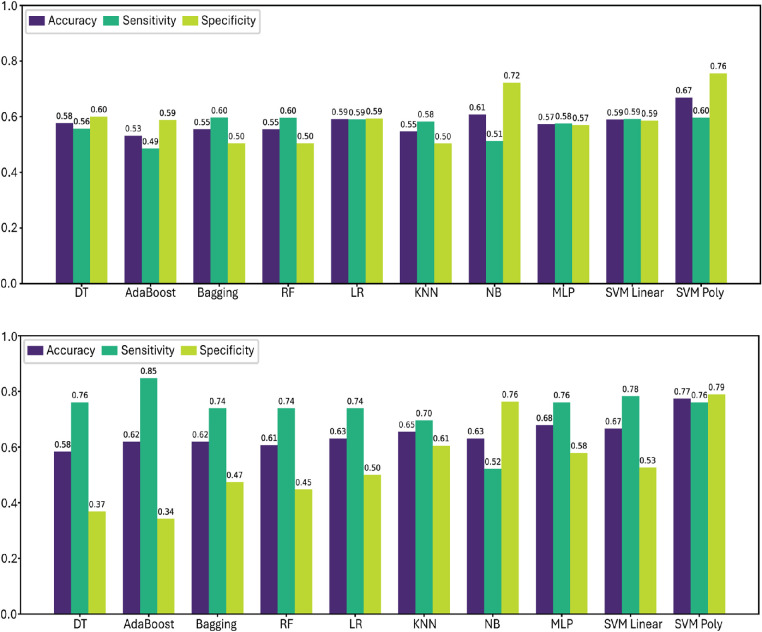
**Performance of** **10** **machine learning models for Parkinson's disease classification based on microsaccade features.** *Top panel*: Results for microsaccade-level classification. *Bottom panel*: Results for subject-level classification, based on aggregated microsaccade-level predictions. Different colors represent the performance metrics: accuracy, sensitivity, and specificity.

Figure 5 illustrates the classification performance of all trained models on both the microsaccade and subject level. In general, all models perform better than a random baseline, achieving over 50.0% accuracy at both the microsaccade and subject levels. The SVM-Poly consistently outperforms all other models, achieving 66.9% accuracy on the microsaccade level and 77.4% on the subject level. Notably, SVM-Poly is the only model to demonstrate comparatively high subject-level performance with a well-balanced sensitivity–specificity trade-off (76.1% and 78.9%, respectively), indicating a reasonable balance between identifying individuals with PD and avoiding misclassification of healthy individuals.

Table 2 illustrates the advantage of SVM-Poly over the other models at both the microsaccade and subject levels. At the microsaccade level, SVM-Poly improves accuracy by an average of 10 percentage points; at the subject level, the improvement is 13 percentage points on average. SVM-Poly performs significantly better than all other models at the microsaccade level, and significantly better than all other models except MLP at the subject level. Importantly, however, MLP shows a substantially less balanced trade-off between sensitivity and specificity compared with SVM-Poly.

**Table 2.** Performance of SVM-Poly Relative to Nine Other Machine Learning Models

	Microsaccade Level				Subject Level			
Models	Acc. %	Δ, (*P* Value)	Sens. %	Spec. %	Acc. %	Δ, (*P* Value)	Sens. %	Spec. %
DT	58	↑ 9 (<0.001)	56	60	58	↑ 12 (<0.001)	76	37
AdaBoost	53	↑ 14 (<0.001)	49	59	62	↑ 15 (0.007)	85	34
Bagging	55	↑ 12 (<0.001)	60	50	62	↑ 15 (0.007)	74	47
RF	55	↑ 12 (<0.001)	60	50	61	↑ 16 (0.004)	74	45
LR	59	↑ 8 (<0.001)	59	59	63	↑ 14 (0.004)	74	50
kNN	55	↑ 12 (<0.001)	58	50	65	↑ 12 (0.02)	70	61
NB	61	↑ 6 (<0.001)	51	72	63	↑ 14 (0.001)	52	76
MLP	57	↑ 10 (<0.001)	58	57	68	↑ 9 (0.11)	76	54
SVM-Linear	59	↑ 8 (<0.001)	59	59	67	↑ 10 (0.035)	78	53
SVM-Poly	67	–	60	76	77	–	76	79

Results are reported at both the microsaccade and subject levels. Acc., Sens., and Spec. denote accuracy, sensitivity, and specificity, respectively. Δ indicates the percentage-point difference in accuracy between the SVM with polynomial kernel (SVM-Poly) and the respective comparison model. Values in parentheses denote *P* values from McNemar's test, indicating the statistical significance of performance differences.

**Figure 6.**
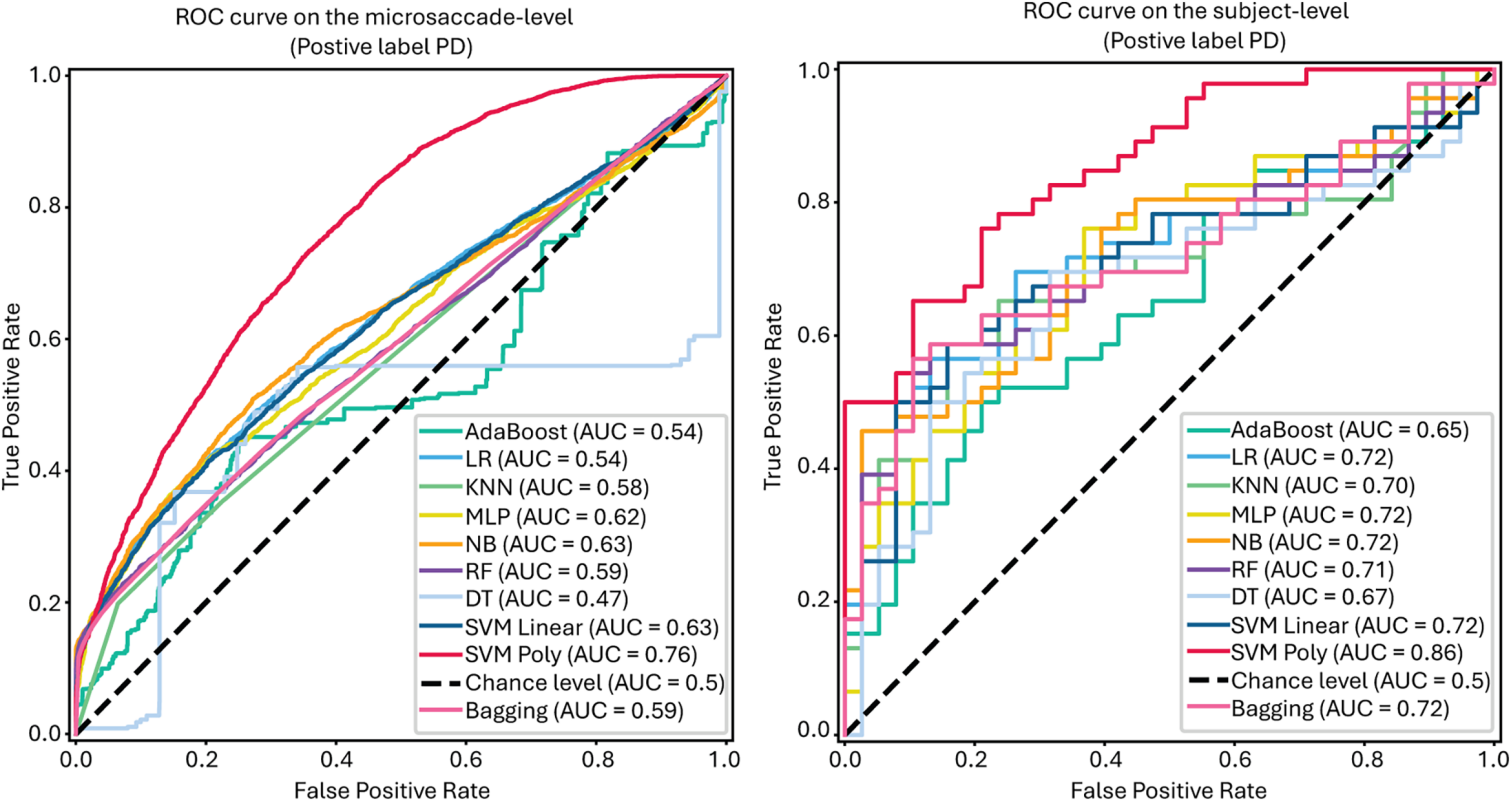
**Receiver operating characteristic (ROC) curves of** **10** **machine learning models for Parkinson's disease classification based on microsaccade features.** *Left panel*: ROC curves computed at the microsaccade level. *Right panel*: ROC curves aggregated at the subject level. Each curve is color-coded by model. The area under the curve (AUC) for each model is reported in the *lower-right corner*. The *dashed diagonal line* denotes chance-level performance (AUC = 0.5).

Figure 6 demonstrates the ROC-AUC for each model on the microsaccade and subject levels. With the only exception of DT, all classifiers exceeded the chance-level ROC-AUC of 0.5 at both microsaccade and subject levels. Subject-level predictions consistently yield higher ROC-AUC values across all models, indicating the effectiveness of aggregating microsaccade-level predictions. SVM-Poly achieves the highest score among all models, with ROC-AUC values of 76.4% at the microsaccade level and 85.7% at the subject level.

### Discarding Predictions With High Uncertainty

**Figure 7.**
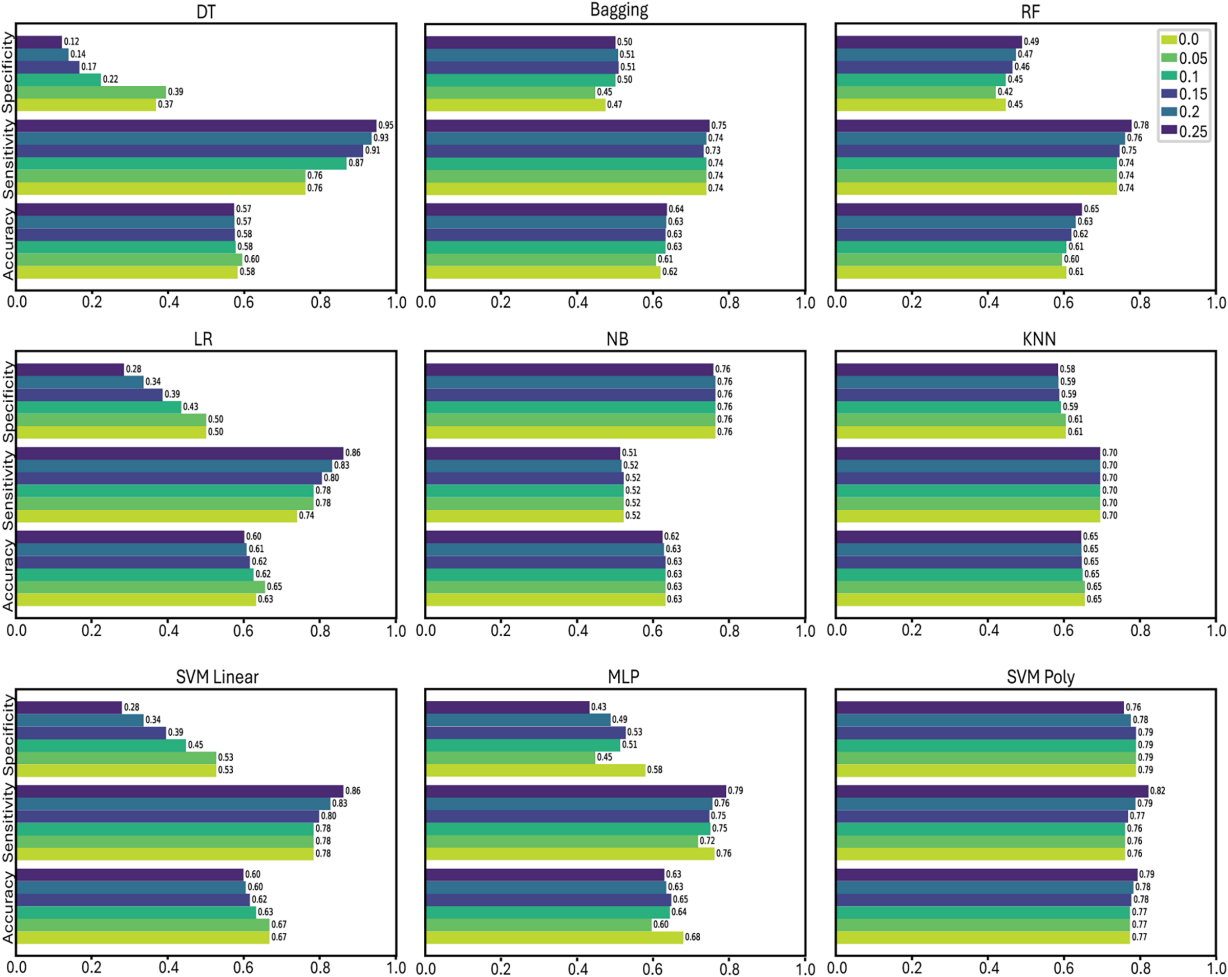
**Performance of nine machine learning models for Parkinson's disease classification across different thresholds for discarding low-confidence predictions.** Results are shown at the subject level, with each subplot corresponding to a different algorithm. Bar colors denote the discard threshold (α = 0.0–0.25, step size = 0.05). The baseline condition (α = 0.0) represents performance without confidence-based filtering. Performance metrics (y-axis): accuracy (*bottom*), sensitivity (*middle*), and specificity (*top*).

Results for five different confidence thresholds applied to nine different algorithms are shown in Figure 7. AdaBoost was excluded from these experiments due to its inferior performance relative to the other ensemble methods in the standard evaluation. Although the DT yielded the lowest performance overall, it was retained to maintain methodological diversity in the model comparison. SVM-Poly consistently achieves the highest performance among all models. At the discard rate of 0.25, its performance surpasses the baseline (α = 0), achieving an accuracy of 79.3%, with a sensitivity of 82.2% and a specificity of 75.8%. For SVM-Poly and RF, accuracy increased with higher discard rates, indicating improved subject-level performance as low-confidence predictions were removed. In contrast, kNN and NB showed little to no effect, with performance remaining essentially unchanged across discard rates. This suggests that kNN and NB inherently produce high-confidence predictions, making them less sensitive to confidence-based filtering. Conversely, other models showed a decline in performance with increasing discard rates. In particular, LR and SVM-Linear exhibited a gradual decrease in accuracy, with SVM-Linear declining from 66.7% to 60.4%. Additionally, DT, LR, and SVM-Linear display a sharp decline in specificity (SVM-Linear decreases from 52.6% to 27.9%) with increasing discard rates. This phenomenon may be attributed to inherent biases in linear decision boundaries when applied to the nonlinear distribution of microsaccade features. The dramatic decrease in specificity suggests that microsaccades correctly classified as HCs tend to receive lower confidence scores, whereas PD classifications are assigned higher confidence scores. This discrepancy may be explained by an imbalance in the distribution of microsaccades, as the microsaccade rate is higher in the PD group than the HC group. To further investigate this possibility, we performed additional experiments balancing the rate of microsaccades between the two groups.

### Class Imbalance

**Figure 8.**
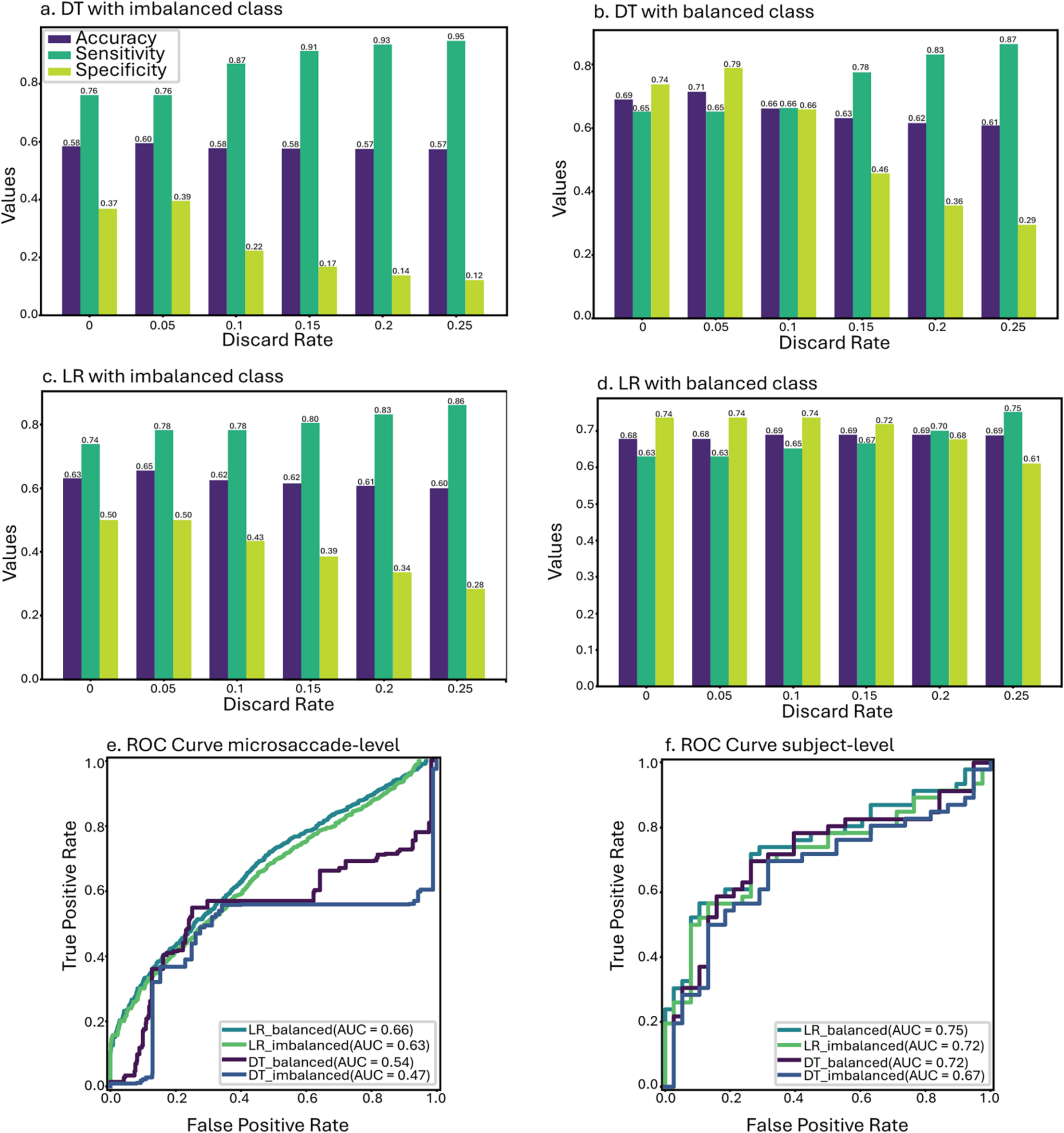
**Performance metrics and ROC curves for decision tree (DT) and logistic regression (LR) models under class-balanced and class-imbalanced conditions for Parkinson's disease classification.** (**a,** **b**) Subject-level performance for DT with imbalanced and balanced classes, respectively. (**c,** **d**) Subject-level performance metrics for LR with imbalanced and balanced classes, respectively. (**e,** **f**) Microsaccade-level and subject-level ROC curves comparing balanced and imbalanced settings for both models, respectively. For **a** to **d**, the x-axis shows discard rates for low-confidence predictions, and the y-axis shows classification accuracy, sensitivity, and specificity.

DT and linear models such as LR are known to be influenced by class imbalance, often favoring the majority class, which in this case results in higher sensitivity. To address this issue, we applied class weighting to DT and LR to evaluate its impact on performance. Figure 8 illustrates the improvements achieved by balancing class distributions, presenting changes in performance across different discard rates. The results indicate that balancing the class distribution enhances ROC-AUC at both the microsaccade and subject levels. Furthermore, when comparing performance metrics across different discard rates, both models exhibit increased accuracy, whereas the difference between sensitivity and specificity is reduced. These findings suggest that class balancing not only improves overall classification performance but also achieves a more even trade-off between sensitivity and specificity.

### Demographic Baseline

To assess the extent to which classification performance could be explained by demographic factors alone, we evaluated a baseline model by training the best-performing model (SVM-Poly) using only the age and sex of participants as predictors. This demographic baseline achieved an accuracy of 63.1% (sensitivity = 65.2% and specificity = 60.5%). The corresponding microsaccade-based model achieved an accuracy of 77.4% in the standard evaluation (sensitivity = 76.1% and specificity = 78.9%). When low-confidence predictions were excluded at α = 0.25, accuracy increased to 79.3% (sensitivity = 82.2% and specificity = 75.8%). Age and sex were not included as predictors in the microsaccade-based models, and the observed performance difference thus reflects the contribution of microsaccade features alone. Overall, the best microsaccade-based models in our evaluation improve upon the demographic baseline by approximately 15 percentage points in accuracy, with corresponding gains of about 14 and 17 percentage points in sensitivity and specificity, respectively.

### Feature Importance

**Figure 9.**
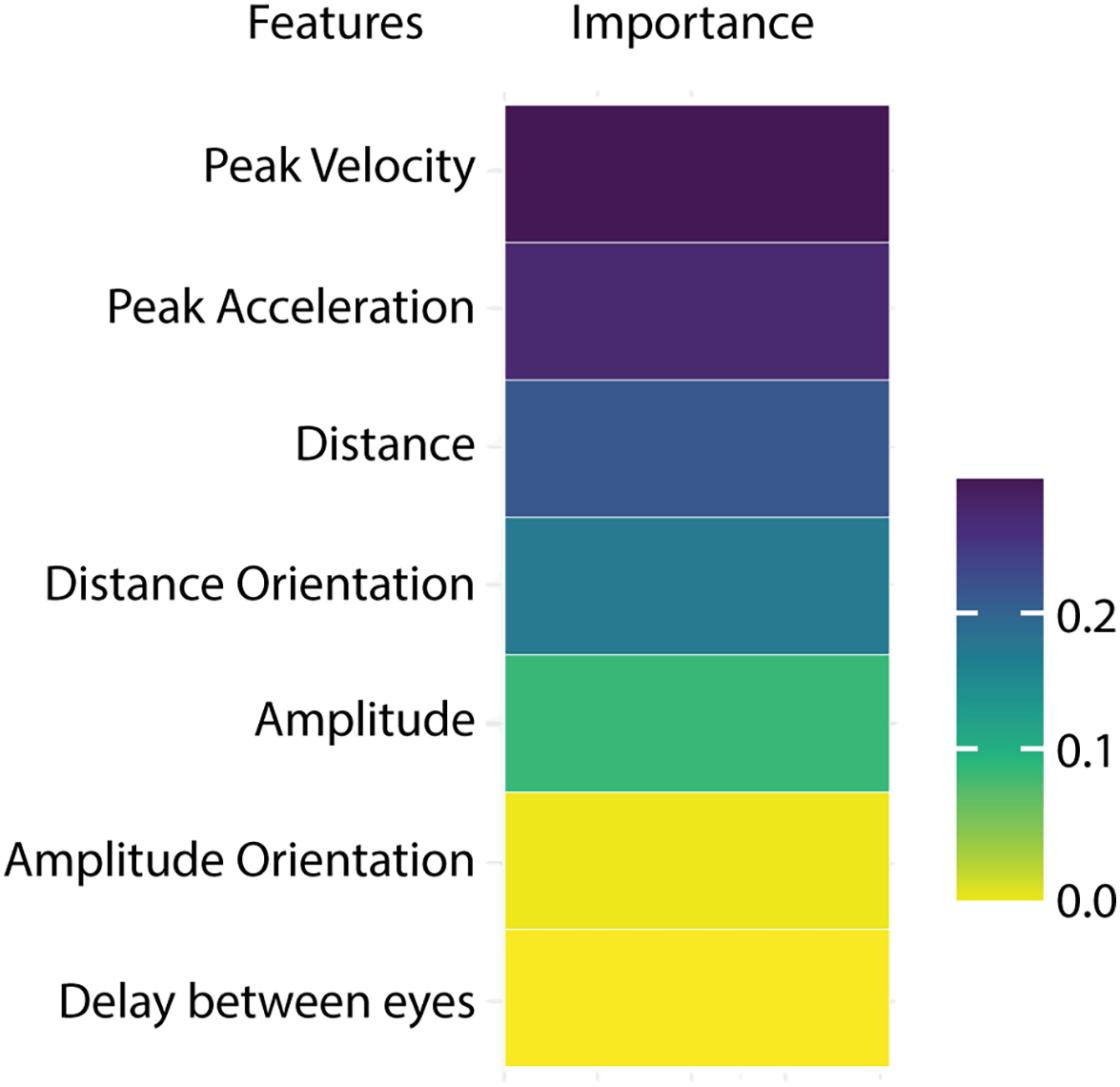
**Importance of microsaccade features in Parkinson's disease classification using a polynomial-kernel SVM.** Scores are normalized to sum to unity, representing each feature's relative contribution to the model's predictive decisions. Darker colors correspond to higher relative contribution.

To shed some light on which microsaccade features contributed most strongly to the classification results, we analyzed the relative contribution of different microsaccade features. Figure 9 illustrates the feature importance of the best-performing model (SVM-Poly). The three features that contributed most strongly to distinguishing PD from HCs were peak velocity, peak acceleration, and the magnitude of the onset–offset displacement (distance). The three features that contributed least were the magnitude of the maximum excursion during the microsaccade (amplitude), the direction of the saccade based on the amplitude vector, and the temporal delay between the two eyes at saccade onset.

These results, however, should be interpreted with caution due to dependencies among the different features. Several microsaccade features are correlated (e.g., amplitude and peak velocity), meaning they carry overlapping information. Consequently, the relative contribution of any given feature cannot be interpreted in isolation, because its apparent importance depends on which other, related features are included in the model. In such cases, the contribution attributed to one feature may be reduced if similar information is already captured by another correlated feature.

## Discussion

We set out to address three questions. First, can small fixational saccades, such as microsaccades, be detected during visual fixation under recording conditions that avoid physical constraints, such as the use of a head-and-chin rest, in healthy participants and participants with PD? Second, do microsaccade characteristics, as measured in this context, differ significantly at the group level between individuals with PD and HCs? Third, to what extent do microsaccade features carry predictive information that can meaningfully distinguish individuals with PD from HCs? We found that microsaccades identified under these recording conditions exhibit characteristics consistent with those reported in the literature under more controlled experimental settings.^35^^,^^37^^,^^41^ Microsaccades in our data occur at frequencies of approximately 1 to 2 Hz on average, with amplitudes typically below 1 degree and a predominantly horizontal direction. Microsaccades also follow the expected linear relationship between amplitude and peak velocity. Thus, although we do not wish to claim that the estimates derived from these recordings are free from the influence of noise, head movements, false microsaccade detections, or other artifacts, they nevertheless appear to provide a characterization of microsaccade dynamics that is consistent with previous work.

Differences emerge when comparing participants with PD to HCs. Microsaccades in the PD group occur more frequently, have a broader amplitude distribution with a higher median amplitude, and show a stronger horizontal component and weaker vertical bias compared with HCs. The slightly higher proportion of horizontal microsaccades in the PD group may reflect a higher incidence of microsaccades occurring as part of square-wave jerk coupling, which typically occurs along the horizontal axis and is more common in PD than in healthy individuals, although the prevalence of square-wave jerks is less pronounced in PD than in other parkinsonian syndromes.^42^ In addition, the PD group shows a significantly larger deviation from the main sequence, indicating greater irregularities in the velocity–amplitude relationship. The main sequence is often interpreted to reflect the integrity of the saccadic system, with deviations from the expected pattern potentially indicating neurological dysfunction.^43^^,^^44^ Overall, these results support the notion that saccadic control mechanisms in PD operate with reduced precision, likely reflecting underlying deficits in neural circuits that influence even the smallest eye movements that can be measured with modern noninvasive video-based eye-tracking techniques.

Experiments using machine learning methods further demonstrated that classification models can distinguish individuals with PD from HC at above-chance levels based solely on microsaccade features. Among 10 basic machine learning methods, a polynomial SVM achieved the highest classification accuracy on held-out test participants, with a well-balanced trade-off between sensitivity and specificity. A diverse set of algorithms was evaluated, spanning linear models, tree-based models, ensemble methods, k-nearest neighbors, Bayesian models, support vector machines, and neural networks. Differences in classification performance across algorithms may provide insight into the structure of the data relevant to distinguishing individuals with PD from HCs.

The results indicate that the underlying data distribution separating PD and HCs is intrinsically nonlinear, as evidenced by the superior and well-balanced performance of SVM-Poly. In contrast, linear models such as SVM-Linear and logistic regression exhibit moderate yet balanced performance at the microsaccade level but their subject-level predictions were notably skewed. MLP, a shallow neural network with a single hidden layer, also showed similar behavior. This pattern likely reflects the limited capacity of linear and shallow models to capture non-linear or weakly separable feature distributions, which may hinder their ability to generalize effectively at the subject level.

Additionally, we found that class imbalance, driven by a higher number of PD participants and more frequent microsaccades in PD, affected model performance. This imbalance had an adverse effect on classification results, particularly for DT and linear models, where specificity dropped markedly after excluding low-confidence predictions. This vulnerability is characteristic of such models under class imbalance conditions. To further investigate this effect, we implemented class weight adjustments in both DT and logistic regression models. These modifications led to measurable improvements in ROC-AUC and accuracy, and narrowed the gap between sensitivity and specificity after removing low-confidence predictions, supporting the hypothesis that class imbalance contributed to the observed degradation in performance.

Taken together, the results of this study suggest that microsaccade dynamics should be given greater consideration within the broader context of eye movement-based biomarkers for PD. Unlike experimental paradigms that require participants to initiate or suppress saccadic eye movements toward a peripheral target, such as in the classical antisaccade task,^45^^,^^46^ passive visual fixation tasks impose minimal cognitive demands beyond basic attentional control. Microsaccades are not volitionally triggered but occur spontaneously during fixation as part of the oculomotor system's natural ongoing activity, which serves both to stabilize the retinal image and prevent rapid adaptation of retinal receptors in response to constant visual input. Altered microsaccade dynamics in PD may therefore reflect early-stage disruptions in oculomotor control, potentially preceding higher-level deficits in inhibitory control and other cognitive functions. This is particularly relevant because, although cognitive symptoms can emerge in the early stages of PD, they typically follow the onset of hallmark motor features such as resting tremor or rigidity and are generally mild during the initial phase of the disease.

That said, the role of attention and cognition in a simple passive fixation task should not be underestimated. Importantly, tasks that do not strongly engage executive control may be particularly susceptible to attentional fluctuations and mind-wandering,^47^^,^^48^ and previous work has demonstrated that microsaccades can be modulated by top-down factors, such as covert shifts of attention.^36^^,^^49^ These findings suggest that moment-to-moment fluctuations in attentional engagement may influence individual microsaccade patterns. However, with respect to the overall results of this study, it is important to note that both participant groups were recorded under identical task conditions, and that the classification models were evaluated on held-out participants using a LOSO cross-validation procedure. This supports the conclusion that the observed microsaccade patterns generalized beyond the training data and were not driven by idiosyncratic fluctuations in individual recordings.

Future work may investigate the dynamics of involuntary saccades produced during visual fixation alongside established eye movement metrics from voluntary saccade tasks. Such metrics could include error rate, latency, and gain in the antisaccade task, as well as spatial accuracy in the memory-guided saccade task,^50^^,^^51^ to assess the extent to which microsaccade dynamics contribute independently and differentially to the prediction of PD. Whereas eye tracking has long been of interest as a potential method for identifying early signs of motor and cognitive impairment in PD, it remains an open question whether predictive accuracy can be improved by combining oculomotor features across distinct tasks to capture different aspects of impairment at different stages of the disease.

Finally, we acknowledge that this study was limited to a set of relatively simple machine learning algorithms, with the aim of establishing a performance baseline and laying the groundwork for future development. There are several promising directions for extending this work, including improvements in data representation (e.g., capturing temporal dynamics or frequency-domain features), exploration of more advanced algorithms (e.g., deep neural networks, including convolutional architectures to capture complex and non-linear patterns), and techniques to address data scarcity, such as synthetic data generation or transfer learning from larger eye-tracking datasets. Such investigations may offer a path toward more generalizable and robust models, improving classification performance and advancing our understanding of how microsaccade dynamics can contribute meaningfully to the development of eye movement-based markers for early detection of PD.

## Supplementary Material

Supplement 1
