# Supplementary material for: Microsaccade Dynamics During Visual Fixation as Markers for Parkinson's Disease: A Machine Learning Approach
Source: Transl Vis Sci Technol. 2026 Jun 16;15(6):19. doi: 10.1167/tvst.15.6.19 (PMC13281954; doi:10.1167/tvst.15.6.19)
Supplement: Supplement 1 [file tvst-15-6-19_s001.docx]

Supplementary material

Description of machine learning models

- AdaBoost: Adaptive Boosting is an ensemble learning technique that improves weak learners by combining multiple iterations of a base model, which assigns weights to the samples and adapts based on the classification errors. The weak learners are normally decision trees. AdaBoost improves accuracy iteratively by assigning higher weights to misclassified samples, so that the weak model trained in the next iteration is fitted to reweighted data. The final prediction is based on a weighted majority vote across all weak models.
- Bagging: Bootstrap aggregating is an ensemble learning method that reduces variance by training multiple weak models on different bootstrap samples of the dataset and averaging their predictions. Each weak learner is trained on a random subset drawn from the training data, whose size is the same as the original dataset but contains duplicate samples. For classification, the predictions from all weak models are combined by majority voting.
- RF: Random forest is an extension of bagging with a mechanism of randomness in feature selection. It builds multiple decision trees and combines their predictions to improve accuracy and reduce overfitting. Like bagging, RF is trained using bootstrap samples of the data. However, at each tree split, only a random subset of features is considered.
- DT: A decision tree recursively splits the dataset into smaller groups based on feature values, forming a tree-like structure. Classification is based on the majority class in the final leaf node reached by a given sample.
- LR: Logistic regression learns a linear relationship between the input features and the probability of class membership by computing a weighted sum of the features. This sum is passed through a sigmoid function to compute the probability of the positive class.
- kNN: K-nearest neighbor predicts the label based on the majority class of its *k* nearest neighbors. The default distance metric used in measuring the distance between points is Euclidean distance.
- NB: Naïve Bayes is a probabilistic classifier based on Bayes' theorem with the assumption that features are conditionally independent given the class label.
- MLP: A multilayer perceptron is a basic artificial neural network that consists of multiple layers of neurons. It includes an input layer, one or more hidden layers, and an output layer. Each neuron in the hidden layers applies an activation function, allowing the model to capture non-linear relationships.
- SVM: Support vector machine finds the optimal hyperplane that best separates the data into classes. A kernel function can map the data into a higher-dimensional space, where it may be easier to find a hyperplane that separates the classes. In this work, we used a linear kernel and a polynomial kernel.
